# Assessing the reliability of an integrated device measuring tongue pressure and bite force among dentate individuals – An observational study

**DOI:** 10.4317/jced.62517

**Published:** 2025-03-01

**Authors:** Jayaraja Banuchandar, Arun Aishwarya, Mani Uma-Maheswari, Mohamed Kasim

**Affiliations:** 1 MDS. Post Graduate Student, Department of Prosthodontics, Sri Ramachandra Dental College and Hospital, Sri Ramachandra Institute of Higher Education and Research, SRIHER (DU); 2 Post Graduate Student, Department of Prosthodontics, Sri Ramachandra Dental College and Hospital, Sri Ramachandra Institute of Higher Education and Research, SRIHER (DU); 3 MDS. Associate Professor, Department of Prosthodontics, Sri Ramachandra Dental College and Hospital, Sri Ramachandra Institute of Higher Education and Research, SRIHER (DU); 4 MDS. Professor, Department of Prosthodontics, Sri Ramachandra Dental College and Hospital, Sri Ramachandra Institute of Higher Education and Research, SRIHER (DU)

## Abstract

**Background:**

Tooth loss leading to complete edentulism negatively impacts quality of life and oral function. Reduced biting force capability affects normal oral function in those who have lost teeth. Decreased tongue pressure significantly affects total oromotor function, especially swallowing. While devices exist to measure biting force and tongue pressure independently, integrated evaluation tools are lacking. This emphasizes developing a reliable integrated tool for simultaneous evaluation. Purpose: To evaluate the reliability of an integrated device designed to measure tongue pressure and bite force simultaneously in dentate individuals.

**Material and Methods:**

Study participants were 80 dentate individuals aged 21 to 55 years who were apparently normal. Participants with specific dental restorations, temporomandibular joint disorders, musculoskeletal disorders, or pacemakers were excluded. The MD30-60 force-sensitive sensors were employed to collect data on tongue pressure and bite force. Three recordings of the tongue pressure and bite force was done by three investigators at 1 minute time interval to prevent muscle fatigue. Statistical analysis utilized Intraclass Correlation Coefficient (ICC) and Lin’s Concordance Correlation Coefficient (CCC) to assess the reliability of measurements.

**Results:**

The analysis of the Intraclass correlation and Lin’s concordance correlation coefficient demonstrated excellent reliability for bite force measurements, with ICC values ranging from 0.882 to 0.906 and a mean bite force of approximately 510 N. Intraclass Correlation Coefficient (ICC) values ranged from 0.795 to 0.827 indicating good consistency among investigators. Bland-Altman analysis confirmed minimal bias and strong agreement for both measurements across investigators.

**Conclusions:**

The novel integrated device demonstrated high reliability for measuring bite force and tongue pressure simultaneously.

** Key words:**Bite force, tongue pressure, reliability, integrated device, oral function, oro-motor function, clinical diagnosis, treatment planning, edentulism.

## Introduction

Complete edentulism has a negative influence on a person’s quality of life and oral function (1). The ability of those who have lost teeth to maintain normal oral function is impacted by their reduced biting force capability (2).

Aycicek *et al*. demonstrated that the result of tooth loss has been associated with a decrease in masseter muscle thickness which is linked to a decrease in chewing ability and occlusal force, resulting in chewing difficulties and an increased risk of malnutrition.(3) Raadsheer *et al*. have demonstrated a strong correlation between edentulism and a concomitant reduction in masticatory efficacy and overall oral function (4,5). Tashiro *et al*. stated that loss of teeth also results in decrease in tongue pressure which may have a significant effect on total oro-motor function, especially on vital physiological functions like swallowing (6). Techniques are needed to understand the relationships between tongue pressure and bite force, as they show a positive correlation with muscle strength. Any changes in these parameters impacts overall orofacial motor function (5).

There are currently a number of devices that can measure different parameters, including biting force and tongue pressure, independently, like the four predominant devices used for assessing oral and swallowing function include the Kay Swallowing Workstation (KSW), Madison Oral Strengthening Therapeutic (MOST), Iowa Oral Performance Instrument (IOPI), and Oro Press device.The IOPI is favoured in research for its ease of use and portability but suffers from poor sensor stability, leading to potential measurement inaccuracies, with no established studies indicating its validity or inter-rater reliability (7).

Several commercially available bite force recording devices include the Dento force 2, a strain gauge device with real-time displays but limited portability and user-friendliness. The IDDK uses a digital dynamometer for precise readings but may have limitations in user comfort during prolonged use. GM10, although portable and easy to use, has potential accuracy issues due to its hydraulic mechanism. The T Scan system, while useful for occlusal analysis, may yield unreliable bite force measurements due to sensor flexibility issues. Lastly, the Dental Pre-scale system provides reliable occlusal contact data but it is time-consuming and cannot perform continuous measurements (8). Integrated evaluation tools that can concurrently assess both tongue pressure and bite force are lacking (7). This limitation in the available diagnostic tools emphasizes the necessity of developing a reliable, integrated tool that can evaluate tongue pressure and biting force simultaneously.

By understanding the relationship between tooth loss, biting force, tongue pressure, and swallowing performance, evaluations of oro-motor function and comprehensive treatment plans for patients with poor oral health can be achieved from this integrated approach. The aim of the study was to assess the reliability of the integrated device designed to measure the tongue pressure and bite force simultaneously in the dentate individuals.

## Material and Methods

The study was conducted in Department of Prosthodontics at Sri Ramachandra Dental College in Porur, Chennai, to evaluate the reliability of the integrated device that records biting force and tongue pressure concurrently. The Institutional Ethics Committee granted ethical approval under reference number CSP-III/24/MAR/03/117. Informed consent was obtained from all study participants.

Sample size calculation:

The sample size calculation was performed using stata statistical software, version 17 (Stata corp., college station, Texas, USA). After assigning the power of the study as 90% and alpha error as 5%, the effective sample size for the study was computed to be 29 using one-sample correlation Fisher’s z test (study parameters: alpha error=0.05, power=0.90, r0 = 0.89, ra = 0.65, delta= - 0.24).

Inclusion and exclusion criteria: 

80 healthy male and female dentate participants aged between 21 and 55 years who were apparently normal were recruited as study participants. The study excluded participants who had a history of temporomandibular joint issues or any musculoskeletal diseases, full coverage restorations, missing dentition or pacemaker implantation.

Description of the device:

The MD30-60 force-sensitive sensors were employed in a tongue force monitoring system to measure tongue pressure and bite force. These sensors operate on the resistive principle, whereby the application of force results in a change in their electrical resistance. Specifically, when a force is applied by the tongue, the internal resistance of the sensors decreases proportionally. With a sensitivity threshold of less than 200 grams, the MD30-60 sensors can detect even the slightest variations in force, providing accurate measurements.

The resistance of the sensors was calibrated to generate an output signal that correlates with the applied force, typically using a standard test voltage of DC 3.33V. This output was sent to an Arduino UNO, which functioned as a central processing unit for the system. The Arduino reads the analog signals produced by the sensors and converted them into digital values suitable for further processing. This enables the system to display the measured force in real time on an LCD screen. The monitoring system is designed for rapid response, featuring a reaction time of less than 1 millisecond and a recovery time of under 15 milliseconds.

The MD30-60 sensors used in this study can accurately measure a wide range of tongue forces, with a working range stretching from 0 to 30 kilograms. Furthermore, they are highly durable, capable of enduring more than a million load cycles, which ensures reliable long-term performance. To facilitate user interaction and data monitoring, the Arduino UNO manages connections with various external modules, including Wi-Fi and Bluetooth components. This allows for wireless data transmission and remote monitoring, enhancing the system’s usability and accessibility for those needing to observe tongue pressure dynamics continuously.

**Figure 1:**
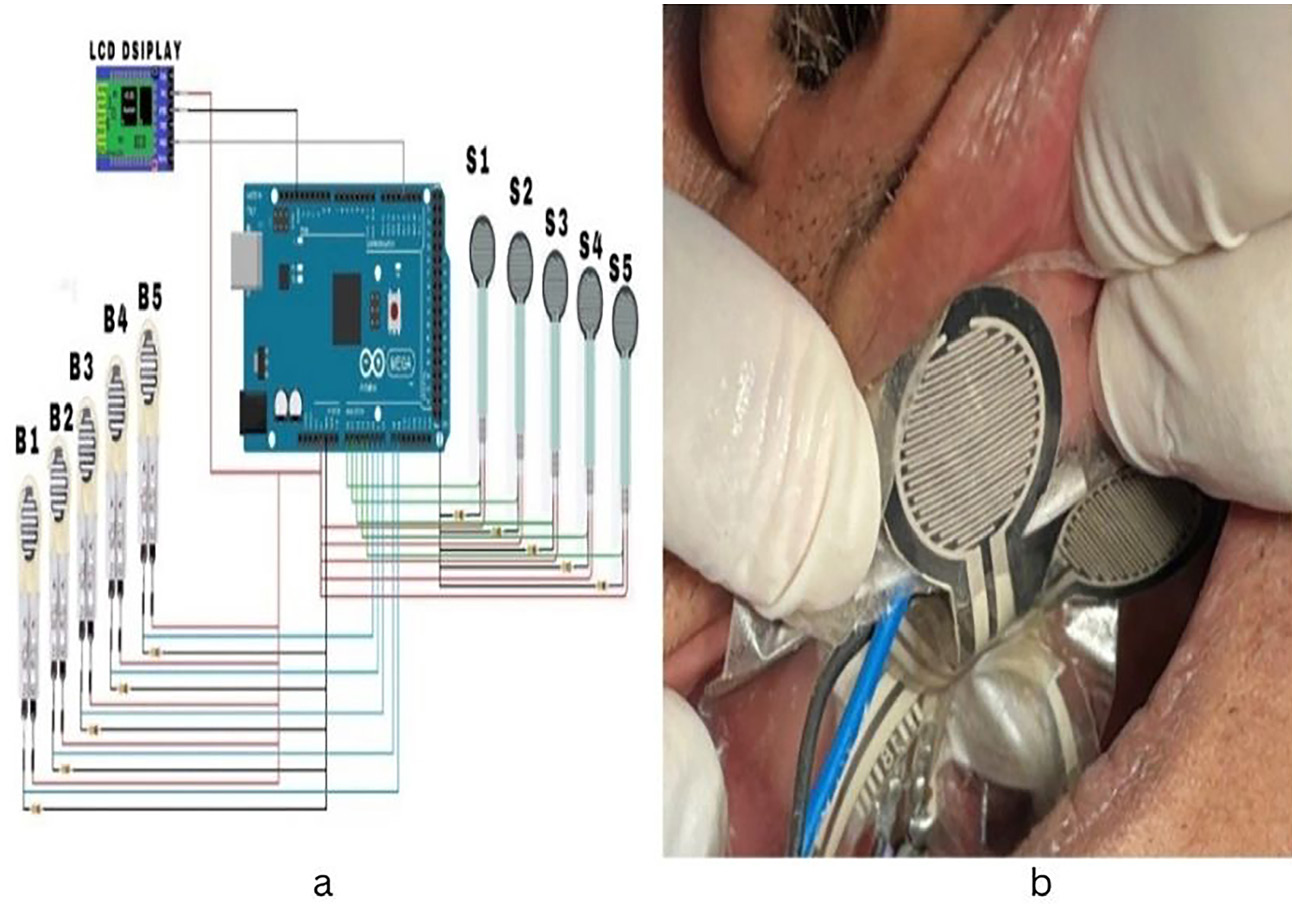
(a) An integrated system with tongue pressure (S1, S2, S3, S4, S5) and bite force measuring sensors (B1, B2, B3, B4, B5) and (b) Placement of pressure sensors against the palate for recording tongue pressure.

The tongue force monitoring system combines the sensitive MD30-60 sensors with the versatile Arduino UNO to provide accurate, real-time measurements of tongue pressure and bite force, supported by robust performance and connectivity features for efficient data management as showed in Fig. 1(a).

Study procedure:

The study participants were seated in an upright position with no support for their head. The pressure sensors were placed against the palate to measure the tongue pressure as given in the Fig. 1(b). At the time of recording the participants were instructed to relax completely, and patient asked to swallow the saliva with pressing the tongue against the palate.

**Figure 2:**
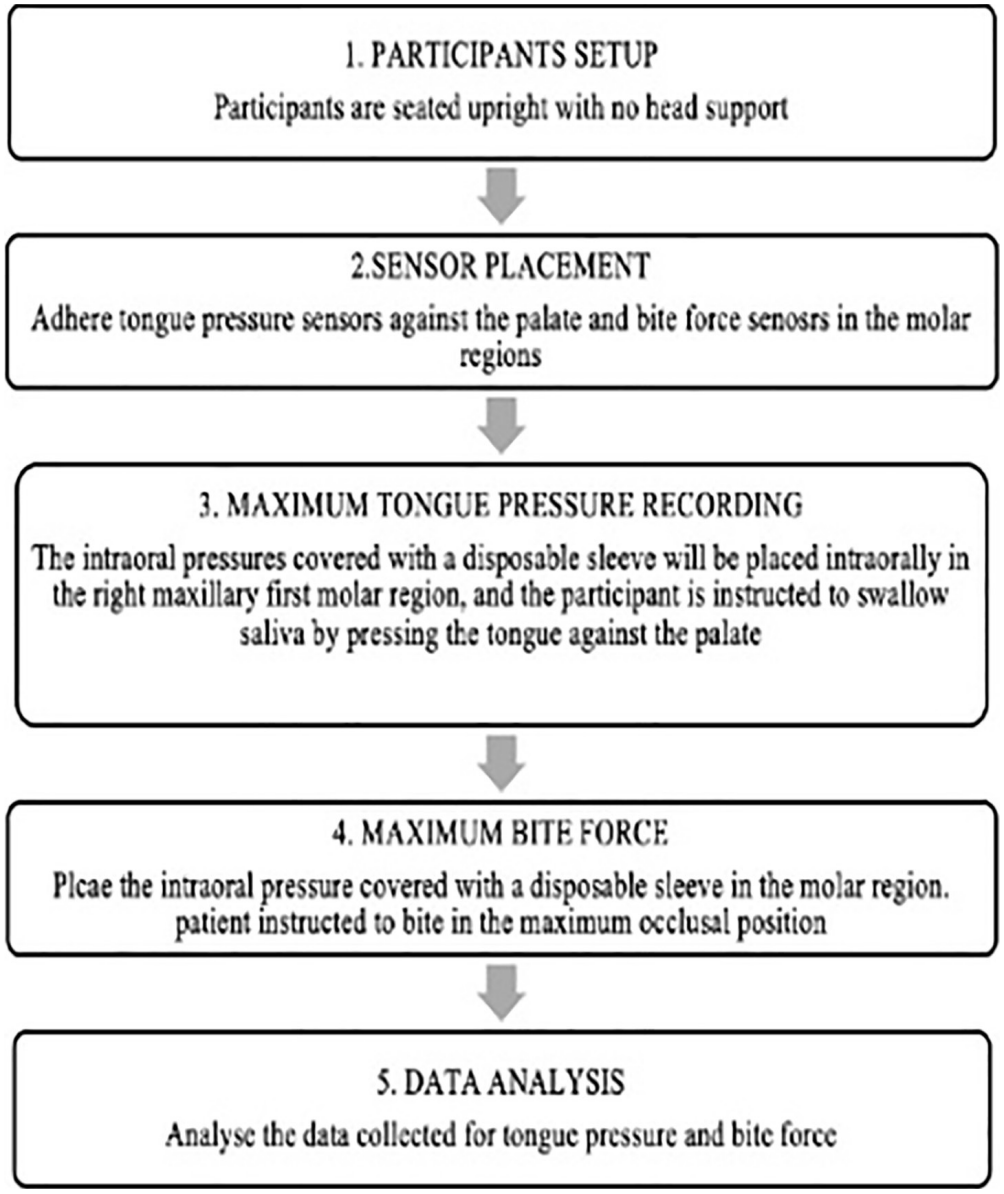
Work flow chart-Step by step process from patient setup to data collection and analysis.

For recording the maximum tongue pressure (MTP), The intraoral pressures covered with a disposable sleeve was placed intraorally adhered to the palate using tray adhesives in the right maxillary first molar region, and the participant is instructed to swallow saliva by pressing the tongue against the palate. Three recordings of the MTP was recorded with one minute gap between each recording to prevent muscle fatigue. Similarly, the participants were asked to swallow again in the same manner, and three recordings of the MTP will be taken. To measure the MBF, intraoral pressure sensors covered with disposable sleeves were placed intraorally in the right maxillary first molar region. The participants were instructed to occlude in the maximum intercuspation position. Three recordings of the MBF were taken, with a one-minute gap between each recording to prevent muscle fatigue. Similarly, the participants were asked to occlude maximally in the occlusal position and three recordings of the MBF were taken. The workflow of the step-by-step process, from participant setup to data collection and analysis, is depicted in Figure 2.

Statistical methods:

The statistical analysis was performed using Stata (Stata Corp, College Station, Texas, USA, version 17). Lin’s concordance correlation coefficient was computed to assess the degree of concordance between observers. Lin’s concordance correlation coefficient was calculated as a product of accuracy and precision (r) parameters, with paired T-tests used for the computations. Accuracy represented the closeness of the data’s reduced major axis to the concordance line, while precision (r) indicated the tightness of the observations around the data’s reduced major axis. Bland-Altman plots were generated to assess the levels of agreement between measurements from the observers. Inter-rater reliability between observers was evaluated using the intra-class correlation coefficient (ICC). The criteria proposed by Koo and Lee were applied to interpret the ICC, precision, and accuracy. According to these criteria, values of <0.50 indicate poor reliability, 0.50–0.74 indicate moderate reliability, 0.75–<0.90 indicate good reliability, and >0.90 indicate excellent reliability.

## Results

The bite force measurements and tongue pressure measurement were recorded for study participants. The descriptive statistics reveal consistent measurements between the three investigators for both Bite Force and Tongue Pressure. For Bite Force, the mean values are nearly identical across investigators, ranging from 509.7 N to 510.1 N, with standard deviations of approximately 76 N. These results suggest strong consistency in the measurements, with minimum values around 400 N and maximum values nearing 639 N. Similarly, for Tongue Pressure, the means are closely aligned, ranging from 41.2 kPa to 41.3 kPa, with standard deviations between 8.2 and 8.4 kPa. Although the variability for Tongue Pressure is slightly higher, the minimum values range from 27.7 to 28.3 kPa, and the maximum values range from 56.2 to 58.5 kPa, indicating reliable data collection across investigators as shown in  Table 1.

The Intraclass Correlation Coefficient (ICC) values for Bite Force show excellent agreement between investigators. Individual ICC values range from 0.882 to 0.906, while average ICC values are consistently 1.00 for all comparisons, signifying strong reliability in the measurements. For Tongue Pressure, the ICC values indicate good agreement, with individual ICCs ranging from 0.795 to 0.827. While these values are slightly lower than those observed for Bite Force, the average ICC values of 1.00 demonstrate consistent and reliable measurements among the investigators as shown in  Table 2.

The Lin’s Concordance Correlation Coefficient (ρ_c) analysis supports the ICC findings. For Bite Force, precision values range from 0.873 to 0.894, and accuracy values range from 0.912 to 0.948. Lin’s CCC values are between 0.859 and 0.914, reflecting strong agreement between measurements with narrow confidence intervals. For Tongue Pressure, precision values range from 0.862 to 0.881, and accuracy values range from 0.905 to 0.939. Lin’s CCC values range from 0.865 to 0.915, indicating good agreement, though slightly less consistent than Bite Force. The results highlight minimal bias and strong reliability in measurements for both variables were depicted in  Table 3.

The Bland-Altman analysis confirms the high level of agreement between investigators. For Bite Force, the mean differences are minimal, ranging from -0.028 to 0.089, with standard deviations between 0.475 and 0.64. The limits of agreement are narrow, such as -0.841 to 1.02 for Investigator 1 vs. 2, reflecting strong consistency. Similarly, for Tongue Pressure, mean differences are closer to zero, ranging from 0.004 to 0.012, with standard deviations between 0.182 and 0.246. The limits of agreement, such as -0.352 to 0.36 for Investigator 1 vs. 2, further support the consistency of measurements were presented in  Table 4.

**Figure 3:**
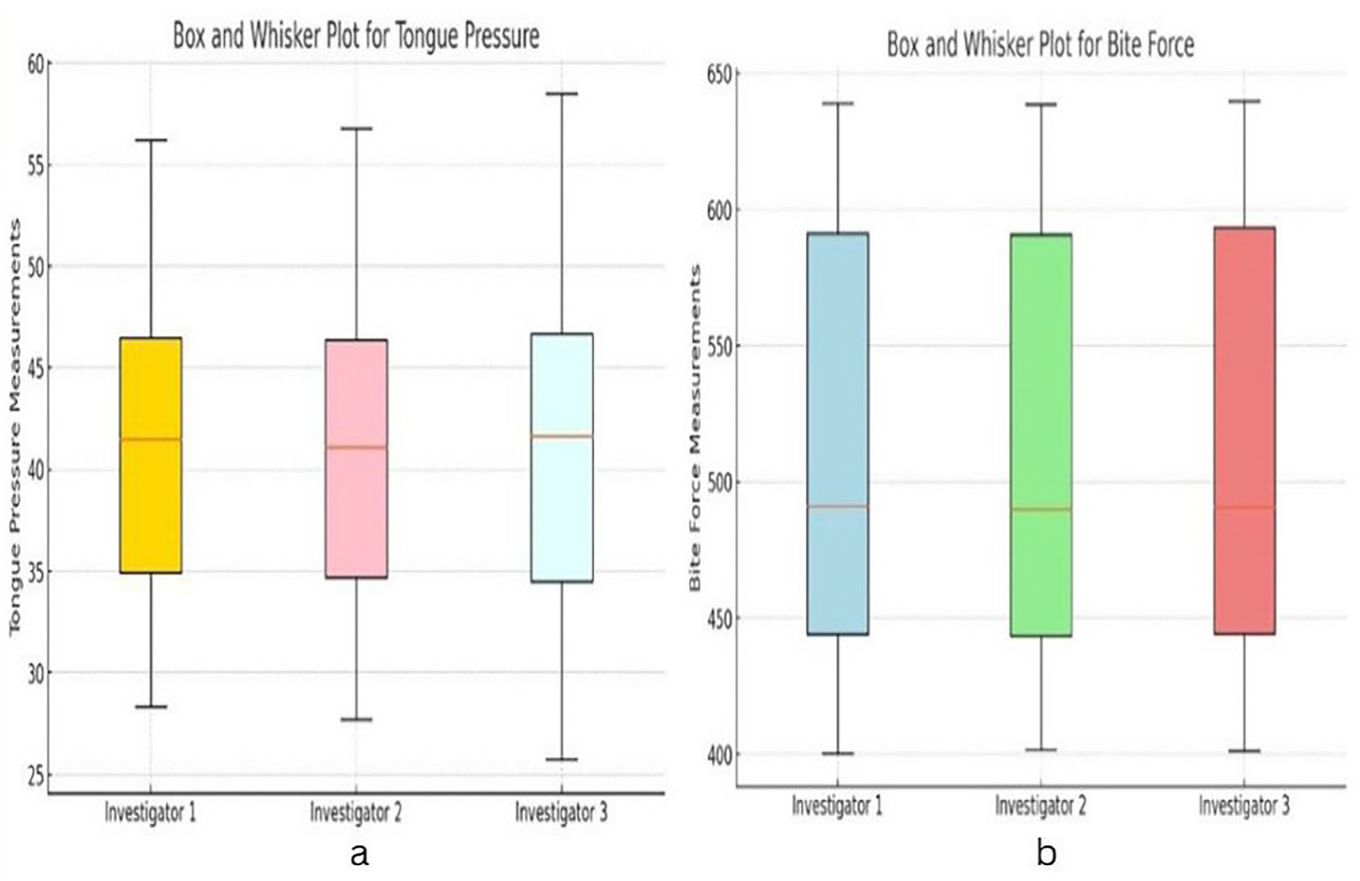
Box and Whisker plot for (a) tongue pressure and (b) bite force.

The box and whisker plots for Bite Force and Tongue Pressure measurements show consistency across the three investigators, with overlapping medians and similar interquartile ranges as shown in Fig. 3(a,b).

**Figure 4:**
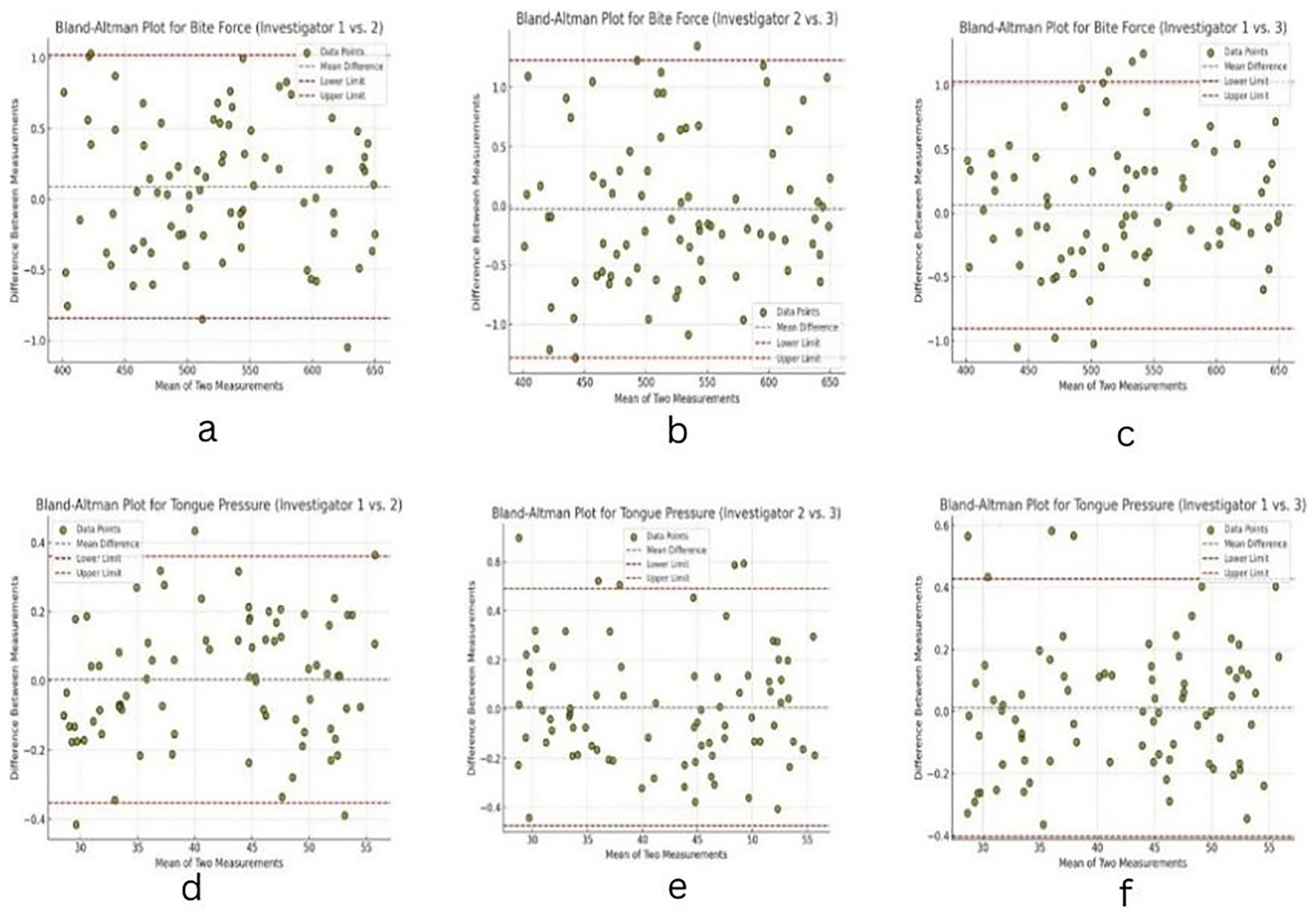
Bland-Altman plot for (a-c) bite force and (d-f) tongue pressure.

The Bland-Altman plots indicate that the differences between measurements are centred around zero with tight limits of agreement, particularly for Tongue Pressure, where the mean differences are minimal. Most of the data points fall within the 95% limits, suggesting no systematic bias between observers. For Bite Force, while variability is slightly greater, the agreement remains excellent, with small mean differences and narrow limits of agreement as displayed in Fig. 4 (a-f).

Overall, the analysis demonstrates excellent agreement for Bite Force and good agreement for Tongue Pressure across all investigators.

## Discussion

Bite force (BF) and tongue pressure (TP) measurements are essential to prosthodontics because they offer important information on oral motor function, treatment results, and general oral health (9). Traditional devices typically assess bite force (BF) and tongue pressure (TP) separately, posing challenges and limiting the clinician’s ability to perform a comprehensive functional assessment. This clinical limitation highlights the need for an integrated, efficient and reliable device capable of simultaneously measuring bite force and tongue pressure (10).

The four most used and studied devices are the Kay Swallowing Workstation (KSW) (11), the Madison Oral Strengthening Therapeutic (MOST) (12), the Iowa Oral Performance Instrument (IOPI) (13) and the OroPress device (14) as mentioned in the  Table 5. Current diagnostic tools for bite force such as the Dentoforce 2 (15), IDDK (16), GM10 (17), T Scan System (18), Dental Prescale System (19), MPX 5700 (20), FSR No. 151 (21), MPM-3000 (22), Flexiforce (23) as mentioned in the Table 5 have several limitations that hinder their clinical utility which includes issues related to portability, sensor accuracy and inability to measure both parameters simultaneously. The novel device presented in this study overcomes the difficulty by combining bite force (BF) and tongue pressure (TP) measurements into a single system.

Bite force serves as a measure of masticatory efficiency and reflects the functional integrity of jaw muscles, occlusal relationships and prosthetic restorations (10). Reduced bite force is common in edentulous patients and those wearing dentures which would eventually lead to compromised chewing efficiency, occlusal instability and diminished quality of life (24). Bite force measurements also help to evaluate the long-term prognosis of prosthetic rehabilitations, including dental implants, fixed restorations and removable prostheses. Whereas, tongue pressure is crucial for swallowing, speech and bolus manipulation during mastication. Reduced tongue pressure can destabilize removable prostheses (25). Simultaneous assessment of tongue pressure and bite force enhances the ability of the clinician to identify functional deficits and design comprehensive treatment plan (26). The novel device presented in this study overcomes this difficulty by combining bite force (BF) and tongue pressure (TP) measurements into a single system. Its capacity to provide precise, real-time measurements facilitates a holistic evaluation of oral function, particularly in patients with compromised musculature due to conditions such as edentulism, neuromuscular disorders or following prosthetic rehabilitation.

Reliability refers to the consistency, repeatability and accuracy of measurements obtained from a device across different conditions, users and time points. Ensuring reliability is essential for clinical and research applications, as it validates the trustworthiness of the data produced (27). For bite force and tongue pressure measurements, high reliability is critical to ensure diagnostic accuracy, treatment monitoring and outcome evaluation. This study evaluated the reliability of the integrated device using statistical analyses which included Intraclass Correlation Coefficient (ICC), Lin’s Concordance Correlation Coefficient (ρ_c), and Bland-Altman plots. These methods provided comprehensive evidence for the precision and consistency of the device.

The device demonstrated excellent reliability for bite force measurements, with ICC values ranging from 0.882 to 0.906. The minimal variation in mean values (509.7 N to 510.1 N) across investigators underscores the precision of the device. Bland-Altman analysis showed a high level of agreement, with narrow limits of agreement thereby validating the consistency of bite force measurements. The reliability for bite force can be attributed to the stable and repeatable nature of jaw-closing muscles, such as the masseter and temporalis which generate strong occlusal forces. The device showed significant reliability for tongue pressure measurements with ICC values ranging from 0.795 to 0.827. Lin’s CCC values (0.865 to 0.915) confirmed strong agreement across investigators. Despite factors like fatigue and sensor positioning, the device exhibited sufficient reliability for clinical use. Bland-Altman analysis showed minimal bias and tight limits of agreement, reinforcing the reliability of tongue pressure measurements.

Simultaneous assessment of bite force (BF) and tongue pressure (TP) enables clinicians to evaluate the interdependent relationship between these parameters and develop targeted interventions that enhance patient outcomes.

The device reduces the need for separate tools by combining bite force and tongue pressure assessment thereby improving efficiency and practicality in clinical settings. The rapid response time of the device (<1 millisecond) and ability to detect minute variations in forces enhance diagnostic precision. Wireless connectivity ensures real-time data transmission thereby making the device suitable for chairside applications and remote monitoring. Its compact, user-friendly design reduces challenges which further improves its usability in clinical and research environments.

The findings have significant clinical and research implications. Clinically, the device facilitates comprehensive assessment of oral function, benefiting fields like prosthodontics, restorative dentistry and speech therapy. It allows clinicians to evaluate patients with conditions such as edentulism, temporomandibular disorders and dysphagia, or those patients who are in the phase of post-prosthetic rehabilitation which necessitates routine monitoring of muscle performance and overall oral function. In research, the device can be used a reliable tool for longitudinal studies investigating the effects of interventions on bite force and tongue pressure. It will assist clinicians and researchers to explore the parameters that influence the oral function, particularly in aging and edentulous population .

The strengths of this study include the use of rigorous statistical analyses, standardized protocols and a clinically robust design of the integrated device. The results provide strong evidence for the reliability of the device in measuring bite force and tongue pressure. However, the study has certain limitations. The participants were limited to healthy dentate individuals, which may restrict generalizability to edentulous or partially dentate populations.

## Conclusions

Within the limitations of the study the following conclusion can be drawn. The integrated device for simultaneously measuring bite force (BF) and tongue pressure (TP) demonstrated excellent reliability for bite force and good reliability for tongue pressure. Statistical validation through ICC, Lin’s CCC, and Bland-Altman analysis confirmed the precision and consistency of the device. This innovative tool represents a significant advancement in the assessment of orofacial function, enabling accurate and efficient evaluation of two critical clinical parameters. The device supports comprehensive diagnosis, treatment planning, and monitoring for patients with conditions such as edentulism, dysphagia, and temporomandibular disorders by offering clinicians a reliable method to assess masticatory performance and tongue strength. The future integration of this tool into various clinical and research settings holds significant potential for advancing the assessment and management of oral motor function.

## Figures and Tables

**Table 1:** Descriptive Statistics.

Study variables	Investigator	Mean	Standard Deviation	Minimum	Maximum
Bite force	Investigator 1	509.9	76.4	400.1	638.9
	Investigator 2	509.7	76	401.7	638.5
	Investigator 3	510.1	76.4	401.3	639.6
Tongue pressure	Investigator 1	41.3	8.2	28.3	56.2
	Investigator 2	41.3	8.4	27.7	56.8
	Investigator 3	41.2	8.4	25.7	58.5

**Table 2:** Inter Rater Reliability performed using intraclass correlation coefficient (ICC).

Study variables	Investigators	Domain assessed	ICC	Lower limit	Upper limit	P value
Bite force	Investigator (1) Vs. Investigator (2)	Individual	0.906	0.876	0.936	<0.001**
		Average	1.00	0.98	1.0	
	Investigator (2) Vs. Investigator (3)	Individual	0.882	0.852	0.912	<0.001**
		Average	1.00	0.98	1.0	
	Investigator (1) Vs. Investigator (3)	Individual	0.9	0.87	0.93	<0.001**
		Average	1.00	0.98	1.0	
Tongue pressure	Investigator (1) Vs. Investigator (2)	Individual	0.827	0.797	0.857	<0.001**
		Average	1.00	0.98	1.0	
	Investigator (2) Vs. Investigator (3)	Individual	0.819	0.789	0.849	<0.001**
		Average	1.00	0.979	1.0	
	Investigator (1) Vs. Investigator (3)	Individual	0.795	0.765	0.825	<0.001**
		Average	1.000	0.979	1.0	

**Table 3:** Precision, Accuracy, and Lin’s Concordance coefficient.

Study variable	Comparison	Precision	Accuracy	Lin's CCC (ρ_c)	Lower CI	Upper CI	P value
Bite Force	Investigator 1 Vs. 2	0.882	0.912	0.914	0.884	0.944	<0.001**
	Investigator 2 Vs. 3	0.873	0.948	0.887	0.857	0.917	<0.001**
	Investigator 1 Vs. 3	0.894	0.927	0.859	0.829	0.889	<0.001**
Tongue Pressure	Investigator 1 Vs. 2	0.876	0.905	0.894	0.864	0.924	<0.001**
	Investigator 2 Vs. 3	0.881	0.939	0.865	0.835	0.895	<0.001**
	Investigator 1 Vs. 3	0.862	0.92	0.915	0.885	0.945	<0.001**

**Table 4:** Bland-Altman Statistics.

Study variables	Comparison	Average	S.D	Lower CI	Upper CI
Bite Force	Investigator 1 Vs. 2	0.089	0.475	-0.841	1.02
	Investigator 2 Vs. 3	-0.028	0.64	-1.281	1.226
	Investigator 1 Vs. 3	0.062	0.493	-0.904	1.027
Tongue Pressure	Investigator 1 Vs. 2	0.004	0.182	-0.352	0.36
	Investigator 2 Vs. 3	0.008	0.246	-0.474	0.489
	Investigator 1 Vs. 3	0.012	0.212	-0.404	0.427

**Table 5:** List of bite force and tongue pressure measuring devices as mentioned in the literature.

Author	Device Name	Description
Anil et al. (11)	Kay Swallowing Workstation (KSW)	A computerized system with three sensors allowing multiple simultaneous measurements of tongue pressure during swallowing at different positions on the palate. However, it is not portable and is very expensive. Excellent intra-rater reliability (ICC = 0.92) in a healthy population
J. Ulrich Sommer et al. (12)	Madison Oral Strengthening Therapeutic (MOST)	A portable device featuring four or five sensors in a pliable intraoral piece, allowing for measurements of tongue isometric pressure against the hard palate in five positions. While easy to use, it has not yet demonstrated reliability or validity. Reliability and validity not yet established
Ji-Su Park et al. (13)	Iowa Oral Performance Instrument (IOPI)	The most researched device for measuring isometric tongue pressure against the hard palate. It is portable and easy to use but has poor sensor stability, which can lead to measurement errors. It has shown good inter-rater reliability (ICC > 0.75), but with measurement artifacts. Good inter-rater reliability (ICC > 0.75) but less reliable than others
McCormack et al. (14)	OroPress	Composed of a biomedical interface pressure transducer and a wireless module, this device captures pressure directly at the sensor tongue interface. It is portable, low-cost, and can measure pressure while swallowing, demonstrating good ICC values (ICC = 0.86) for reliability. [7] Good-to-excellent reliability (ICC = 0.86)
Tzakis et al. (15)	Dentoforce 2	A metal fork with a strain gauge transducer, coated with soft rubber for interocclusal placement. Displays bite force (in Newtons) on a digital recorder (Multimeter 4055). Measures forces up to 1000 N with a vertical height of 11 mm.
Kogawa et al. (16)	IDDK	Digital dynamometer with a capacity of 1000 N, with a bite fork consisting of metal rods and plastic disks. Features a "set-zero" key, peak value registration, and can display values in N or Kgf. Vertical height of the fork is 14.6 mm.
Serra et al. (17)	GM10	Hydraulic pressure gauge with a biting element made of vinyl encased in a disposable polyethylene tube. Measures bite force digitally, with a range of 0 – 1000 N and accuracy of ±1 N. Portable and user-friendly.
Kerstein et al. (18)	T Scan System	Computerized occlusal analysis system developed for prosthodontics. Uses a thin sensor to evaluate bite force and occlusal contact area. Provides real-time data; has faced criticism for accuracy issues in measuring bite force.
Inomata et al. (19)	Dental Prescale System	Pressure-sensitive system for measuring bite force, occlusal contact area, and bite pressure. Utilizes colour-forming microcapsules activated by bite force with reliable results, although it can be time-consuming and requires data entry on the same day.
Serra MD et al. (20)	MPX 5700	Consists of a tube and pressure sensor connected to an analog-to-digital converter for bite pressure measurement. Limited to air pressure measurements, with potential bounce and lag time issues.
Gomes et al. (21)	FSR No. 151	A circular conductive polymer pressure-sensing resistor with a diameter of 12 mm. Its resistance decreases with increasing pressure. Used in various bite force studies, providing flexibility and reliability.
Ogura et al. (22)	MPM-3000	Digital multi-meter coupled with an occlusal force transducer, measuring maximum bite force displayed in kg. Diameter of the plate is 17 mm, effectively used in different studies.
Freeman et al. (23)	Flexi-force	Developed for measuring bite force in small mammals, consists of a piezoresistive load cell strip. Can measure up to 4500 N but less accurate compared to other load cells; used successfully in several studies.

## Data Availability

The datasets used and/or analyzed during the current study are available from the corresponding author.
